# Detection of hepatitis E virus (HEV) through the different stages of pig manure composting plants

**DOI:** 10.1111/1751-7915.12064

**Published:** 2013-11-11

**Authors:** M García, S Fernández-Barredo, M T Pérez-Gracia

**Affiliations:** 1 Área Microbiología, Instituto Ciencias Biomédicas, Facultad Ciencias de la Salud, Universidad CEU Cardenal HerreraMoncada (Valencia), Spain; 2 Centro Diagnóstico Veterinario (CEDIVET)Valencia, Spain

## Abstract

Hepatitis E virus (HEV) is an increasing cause of acute hepatitis in industrialized countries. The aim of this study was to evaluate the presence of HEV in pig manure composting plants located in Spain. For this purpose, a total of 594 samples were taken in 54 sampling sessions from the different stages of composting treatment in these plants as follows: slurry reception ponds, anaerobic ponds, aerobic ponds, fermentation zone and composting final products. HEV was detected by reverse transcription polymerase chain reaction (RT-nested PCR) in four (80%) of five plants studied, mainly in the first stages of the process. HEV was not detected in any final product (compost) sample, destined to be commercialized as a soil fertilizer, suggesting that composting is a suitable method to eliminate HEV and thus, to reduce the transmission of HEV from pigs to humans.

## Introduction

Hepatitis E virus (HEV) is the main causative agent of enterically transmitted non-A non-B hepatitis (Purcell and Emerson, 2013; Perez-Gracia and Rodriguez-Iglesias, 2003). Hepatitis E is considered an infectious disease endemic in developing areas such as India, Africa and South-east Asia, because of poor sanitary conditions in drinking water. When it was first reported in developed countries, HEV was related to travel to endemic areas. However, the epidemiology of HEV in industrialized countries like Spain have changed in the last years, with an increasing number of non-travel associated sporadic cases (Perez-Gracia *et al*., 2004). In industrialized countries, hepatitis E actually represents less than 3% of acute viral hepatitis cases (Purdy and Khudyakov, 2011). However, the overall relevance of HEV infection has been underestimated and the disease may to be considered like a global health problem at the present time. The WHO estimates that more than 3 million individuals suffer symptomatic acute hepatitis E and the disease causes around of 70 000 deaths at year (Pischke and Wedemeyer, 2013). The overall mortality of hepatitis E in the general population is less than 1%; but it can reach up to 28% in infected pregnant women (Khuroo and Kamili, 2003).

The high phylogenetic homology observed between pig and humans strains of genotype 3 in the same geographical area suggests that hepatitis E is a zoonotic disease. This statement has been reinforced by the results of several studies in Japan (Takahashi *et al*., 2004), Korea (Ahn *et al*., 2005), the UK (Banks *et al*., 2004; Ijaz *et al*., 2005; Tolari *et al*., 2006) and also in Spain (Pina *et al*., 2000; Perez-Gracia *et al*., 2007), where they reported a nucleotide similarity of 94% between human and pig strains.

Besides the high nucleotide homology observed, HEV has been demonstrated to be able of crossing the species barrier, raising concern for the potential ways of zoonotic transmission from swine to humans (Meng, 2011).

The direct application of the slurries in agricultural areas is the more economic and ecological method in the management and recycling of the same ones (Ziemer *et al*., 2010; Pardo *et al*., 2011), following the parameters established by the law. Nevertheless, the appearance in the last years of zones with high density of cattle intensive developments generates a large amount of wastes. In this sense, Spain is the second country in the business of pig livestock in European Union (EU), with 25 million of units. The area studied has 170 farms distributed in an approximated surface of 1000 square miles, generating a total amount of 1 million of tones of slurries at year (source: EUROSTAT DATABASE, 2009) of 41 million of tones at year generated in EU. The management of these products turns out to be complicated and inefficient, constituting an environmental and sanitary problem because of zoonotic pathogens like HEV, may be transported to drinking water resources (Bolado-Rodriguez *et al*., 2010; Hazam *et al*., 2010; Meng, 2011) and other problems like excessive nitrification of soil and presence of excessive concentration of heavy metals. These problems worry both the livestock sector and Public Health authorities.

In this sense, the pig manure composting plants constitutes an innovative purification system in Spain, and these are the only plants that are currently being used. These procedures performed in these plants are safe alternative methods in the management of the slurries.

The aim of this study was to evaluate the presence of HEV in the different stages of pig manure composting plants.

## Results

Two different types of composting plants (type A and type B), based on the type of manure treatment, were studied. Plants performing a total treatment of the slurry (type A plants), include an initial separation of liquid and solid phases of the slurry by centrifugation and posterior purification by fermentation procedures.

**Fig. 1:**
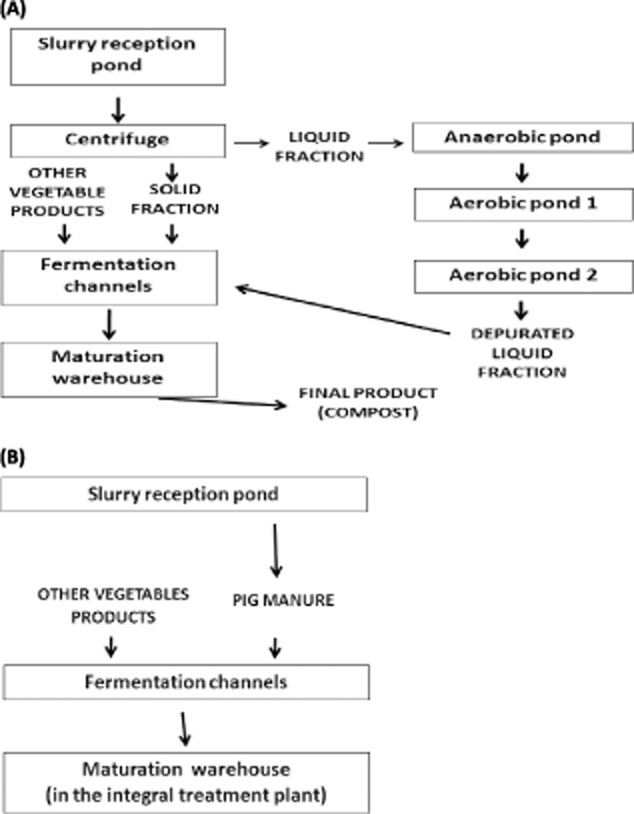
A. Plants type A flow-chart.B. Plants type B flow-chart.

The purification process in type A plants (Fig. 1A) starts with an initial centrifuge. Liquid fraction of the slurries is carried to the anaerobic lagoon, where the slurries will remain 100 days. In this time; methanogenics, acetogenics and hydrolysis processes lead to a reduction of the smell, decrease of pathogens and stabilization of organic matter and hence reduction of BOD (biological oxygen demand).

Liquid from the anaerobic pond goes on later to the aerobic ponds 1 and 2, where the slurries will remain 10 days in each one. In this stage, offensive odours decrease and reduction of pathogen continues, conversion of available nitrogen to ammoniacal nitrogen and a decrease of C : N ratio is performed also.

The final product of this step is a depurated liquid fraction that will be mixed with the solid fraction and other vegetables, and carried to the fermentation channels where the composting process is performed. Composting is an aerobic process that requires a continuous supply of air with mechanical mixing during 21 days approximately. The temperature in the pile can rise in the 10 first days to as high as 65°C.

As opposed to type A plants, the type B plants does not make a fractionated treatment of the slurry (Fig. 1B) and directly use it in the composting process.

The final product is the compost, an inoffensive, depurated and a better soil fertilizer than raw slurries.

We have studied two type A plants (A1and A2) and three type B plants (B1, B2 and B3). A total number of four (80%) of five plants studied were positive to the presence of RNA-HEV. According to the type of plant, HEV was detected in two (100%) of the two type A plants and in two (66.66%) of the three type B plants (Table 1).

**Table 1:** Nested-PCR results according to the number of sampling sessions in the different stages of treatment in type A and B plants

Plant	Reception pond	Anaerobic pond	Solid product from centrifuge	Aerobic pond 1	Aerobic pond 2	Fermentation channel (Start)	Fermentation channel (End)	Maturation warehouse	Final pellet
A1	24/27^a^ (88.89%)^b^	18/27 (66.66%)	3/27 (11.11%)	3/27 (11.11%)	0/27 (0%)	0/27 (0%)	0/27 (0%)	0/27 (0%)	0/27 (0%)
A2	9/9 (100%)	6/9 (66.66%)	6/9 (66.66%)	0/9 (0%)	0/9 (0%)	0/9 (0%)	0/9 (0%)	0/9 (0%)	0/3 (0%)
B1	0/3 (0%)	–	–	–	–	0/3 (0%)	0/3 (0%)	0/3 (0%)	0/3 (0%)
B2	3/6 (50%)	–	–	–	–	0/6 (0%)	0/6 (0%)	0/6 (0%)	0/6 (0%)
B3	3/9 (33.33%)	–	–	–	–	0/9 (0%)	0/9 (0%)	0/9 (0%)	0/9 (0%)

a Sampling sessions HEV positive/total number of sampling sessions.

b Percentage of positive sampling sessions.

**Fig. 2:**
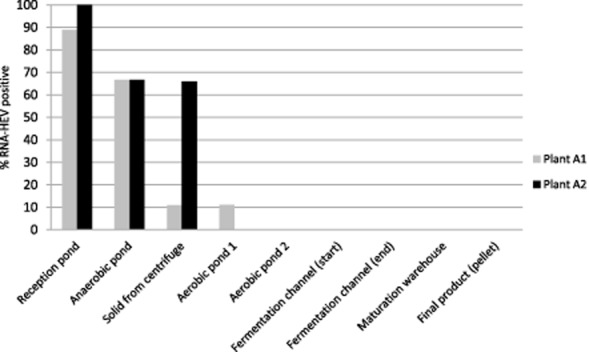
RNA-HEV detection through the different stage of pig manure treatment in type A plants.

According to the different stage of treatment, in plant A1, HEV was detected in 24 (88.89%) of 27 sampling sessions in the reception pond. In 18 (66.67%) of 27 sampling sessions, HEV was detected in the anaerobic pond; which is the first step of purification process. Only in three (11.11%) of the 27 sampling sessions, HEV was detected in the aerobic pond number 1; from this stage forward to the end of the composting process, HEV was not detected (Fig. 2).

In plant A2, HEV was detected in nine (100%) of nine sampling sessions in the reception pond. In six (66.66%) of nine sampling sessions, HEV was detected in the anaerobic pond; after this stage of treatment, HEV was not detected in either solid or liquid samples (Fig. 2).

In type B plants, HEV was not detected in plant B1 and it was detected in three (50%) of six sampling sessions in plant B2, and in three (33.33%) of nine sampling sessions in plant B3 in the respective reception ponds. HEV was not detected in any solid samples in these plants.

## Discussion

In recent years, the increase of animal farming has generated an intensive and megascale livestock operations that produce a large amount of animal wastes worldwide (Cole *et al*., 2000; Spencer and Guan, 2004). A lot of causative agents of many infectious diseases have been identified in slurries, including HEV. The storage and treatment before land application are commonly performed in farms; however, it does not result in a total removal of pathogens from the manure (Ziemer *et al*., 2010).

Pig manure slurry is an emergent health and environmental problem and a potential source of infectious pathogens like HEV (Kasorndorkbua *et al*., 2005). If the slurries do not undergo a process of purification, and are spread without control over crop fields, population can be infected by the virus by consuming contaminated water from aquifers or vegetables irrigated with HEV contaminated water. Moreover, veterinarians, farmers and swine workers have been observed to be at a higher risk (Perez-Gracia *et al*., 2007; Galiana *et al*., 2008), as well as the contact with pig manure constitutes a factor of higher risk. To reduce the impact of animal waste production several technologies have been developed around the world. One of them has been performed in a high swine production zone in Castellon (Spain), with the installation of five pig manure composting plants (A1, A2, B1, B2, B3) with a pioneer treatment system.

Previous works evaluated the survival of pathogens after the treatment of the sludge (Sobsey *et al*., 2003; Costantini *et al*., 2007; Wagner *et al*., 2008), but this is the first study that evaluates the effectiveness of swine manure composting plants in the elimination of HEV.

The HEV is widely widespread in the zone of action of the plants studied (Fernández-Barredo *et al*., 2007). In fact, in this study HEV was detected in 80% of the plants. These data coincide with other works in the same geographical zone (Seminati *et al*., 2008) that reported in some cases 76.19% of HEV prevalence in farms closer to these plants (Fernández-Barredo *et al*., 2006).

In plants with total treatment of the slurries (type A plants), HEV was not detected in any sample from the liquid fraction after the stage of treatment corresponding to the aerobiosis 1 pond (Fig. 1A), suggesting that a high percentage of HEV had been inactivated to this point in anaerobic and aerobic ponds, and reduced its presence at levels not detectable by PCR.

There are no data reporting the resistance of HEV to anaerobic purification processes; however, a work about the resistance of porcine enteric virus of the *Caliciviridae* family at different purification treatments of slurry (Costantini *et al*., 2007) confirmed the reduction of the number of viral particles after subjecting slurry to anaerobic digestion. Additionally, this study confirmed that the aerobic treatment was able to reduce the concentration of more resistant virus, such as rotavirus type A, B and C, which themselves were capable to resist anaerobic processes, to undetectable levels by PCR.

Additionally, the fact of HEV in type A plants was detected in a higher percentage in the reception pond compared with the detection of HEV in solid products from centrifuge, could be due to the lesser resistance of the virus in solid matrices (Pesaro *et al*., 1995).

HEV was not detected in any solid samples of processed product by fermentation. The high temperatures achieved in the fermentation channels (approximately 65°C), could explain the lack of HEV amplification. In this sense, a study performed by Emerson and colleagues in 2005, conducted with human and swine strains, showed that HEV genotypes 1 and 2, were 50% inactivated at temperatures between 45°C and 50°C, and totally inactivated at 60°C. Only Mex 14 (genotype 2) strain was observed to be 100% resistant to 56°C and 20% to temperatures of 60°C (Emerson *et al*., 2005). In addition, a recent study (Barnaud *et al*., 2012), shows an efficient inactivation of the virus when food products from infected by HEV pork livers, were heated at least 20 min to an internal temperature of 71°C.

In this way, the low percentage of HEV positive samples after anaerobic digestion, suggests that large amounts of virus do not exceed this stage of treatment. The lack of detection of HEV in solid samples at the end phases of the composting treatment and in the final product, suggests that composting process is effective to eliminate this virus from the slurries, and thus to reduce the transmission of HEV from pigs to humans.

## Experimental procedures

### Collection of samples of composting plants

A total number of 594 liquid and solid samples were collected in 54 different sampling sessions from March 2006 to December 2011 in five plants distributed as follows (Table 1): two type A plants (A1 and A2) and three type B plants (B1, B2 and B3). The higher number of samples collected from type A plants, 504 (87.5%) is due to the fact that these plants assemble the majority of purification processes and therefore receive more manure than type B plants. In the same way, the higher number of liquid samples, 360 (60.6%) corresponds to the higher number of purification processes carried out in the liquid phase of the slurry.

The plants are located in Eastern Spain, in a zone endemic for porcine HEV (Fernández-Barredo *et al*., 2006). The plants were sampled in 54 different sampling sessions, in which a variable number of samples were taken in each stage taking into account the volume of each pond. Thus, in the anaerobic pond a total number of 180 samples were collected in five different points at each sampling. The pond was considered positive to HEV when at least one of the five sampling points was positive. The rest of the ponds were equipped with a homogenization system which was activated 10 min before the sampling, therefore only one sample of a larger volume was taken. Solid samples were homogenized by mechanical processes included in the periodic aeration system performed in the integrated treatment.

It was impossible to take the same number of samples in type B as in type A plants, due to a premature closing of the same ones because of technical and economical problems.

Liquid samples (50 ml), were collected directly from the ponds and kept in sterile falcon tubes until processing. All the samples were transported refrigerated immediately to the laboratory. Solid samples were diluted at 10% (w/v) in sterile phosphate buffer saline (PBS) pH 7.2.

### Virus particle concentration, RNA extraction and reverse transcription-nested PCR (RT-nested PCR) with internal control

All the samples collected were initially centrifuged 1 min at 1000 *g* and 15 ml of the supernatant were centrifuged at 4°C, 1 h at 3000 *g* to concentrate the virus particles. Remaining supernatant (1.5 ml) was transferred to a sterile eppendorf tube and it was centrifuged at 12 100 *g* for 10 min, and 1 ml of the supernatant was stored at −80°C.

RNA was extracted from 140 μl of each concentrated sample according to the method described by Fernández-Barredo and colleagues (2006). Two pairs of degenerated oligonucleotide primers based on human and swine HEV sequences, were used to amplify a 348-bp-long fragment from the HEV open reading frame 2 (ORF-2) using a reverse transcription-nested polymerase chain reaction (RT-nested PCR) (Huang *et al*., 2002). A negative and a positive control from a naturally infected pig (GenBank Accession No. AY323506) were included in each assay. The different stages of the procedure were performed in different places to avoid the possibility of cross-contamination. The faecal samples may contain RT-PCR inhibition substances like phenolic and methabolic compounds, the concentration and presence of these inhibitors is different and heterogeneous from sample to sample (Rutjes *et al*., 2007). To detect the presence of these substances an internal control was included within RT-PCR reaction. The internal control used is a modified 77 bp PCR product cloned into a plasmid containing a sequence that can be amplified simultaneously with the target using the same primers set in the first PCR performed according to the protocol described by Huang and colleagues in 2002. The 77 bp amplified fragment was detected in the electrophoresis gel. PCR inhibition was not detected. The sensitivity of the PCR was estimated in 31.6 PID_50_ of infectious swine HEV (Huang *et al*., 2002).

The PCR products were separated by electrophoresis in a 2% agarose gel and detected by staining with ethidium bromide (0.5 μg ml^−1^). The samples were considered positive to HEV when a band of 348 bp was seen in the agarose gel. Amplicons from all positive samples were purified and confirmed by sequence analysis. Sequences obtained in this study have been submitted to the GenBank database under Accession No. KC145131–KC145147.
